# Ion Channels and Neurological Disease—2nd Edition

**DOI:** 10.3390/life16061004

**Published:** 2026-06-15

**Authors:** Carlo Musio, Marzia Martina

**Affiliations:** 1 Institute of Biophysics—IBF, National Research Council—CNR, Via Sommarive 18, 38123 Trento, Italy; 2 Human Health Therapeutics Research Centre, National Research Council Canada, 1200 Montreal Road, Ottawa, ON K1R OR6, Canada

## 1. Introduction

Ion channels are essential for regulating ion movement across cellular membranes, a process especially critical within the central nervous system (CNS) where these channels govern membrane physiology and neurotransmission [1]. Dysfunctions in ion channels, arising from genetic mutations or structural changes (channelopathies), are implicated in numerous neurological disorders such as epilepsy, migraine, and pain [2]. In addition, neurodegenerative diseases such as Alzheimer, Parkinson’s, and amyotrophic lateral sclerosis have been shown to exhibit disruptions in neuronal excitability linked to dysfunction in ion channel activities [3]. Research into ion channel mechanisms and interaction with auxiliary proteins is needed to enhance our understanding of their role in neurological disorders and to develop new therapeutic strategies.

## 2. This Special Issue

In the second edition of this Special Issue, “Ion Channels and Neurological Disease”, in *Life*, we expand the subject covered by the first edition [4] regarding dysfunctions of ion channels. In this issue, we present advanced research that integrates structural biology, neurophysiology, and clinical pharmacology. We also included a study on ion transporters. Transporters have a pivotal role in maintaining ionic homeostasis and neuronal excitability, and their dysfunction contributes to numerous neurological diseases, including epilepsy, neurodegeneration, and stroke.

This Special Issue includes three research articles and six reviews; all articles are ordered and described below according to their typology (research and review articles), while they are listed at the end of this editorial according to the journal’s Special Issue website order.

### 2.1. Research Articles

The Na_v_ family comprises nine distinct subtypes, designated Na_v_1.1 to Na_v_1.9 [5], which play a critical role in the initiation and propagation of neuronal action potentials, and their dysfunction is strongly associated with a wide range of neurological disorders and channelopathies, including epilepsy, migraine, neuropathic pain, and neurodevelopmental diseases [5]. In their articles, Arman et al. (Contribution 1) and Plakhova et al. (Contribution 2) focus on the structure and modulation of voltage-gated sodium channels (Na_v_) to identify new targets for the development of therapeutics for neurological disease.

Arman et al. (Contribution 1) investigate the modulation of Na_v_1.2 and Na_v_1.6 by auxiliary proteins. Mutations in the genes encoding Na_v_1.2 channels can lead to conditions such as autism, intellectual disability, and epileptic encephalopathies, while mutations in Na_v_1.6 are associated with developmental and epileptic encephalopathies that often include motor symptoms. Given the severe neurological disorders linked to these channelopathies, understanding how auxiliary proteins modulate channel function is particularly important. The authors describe the interaction between the intracellular fibroblast growth factors (iFGFs) and Na_v_s through protein–protein interactions (PPI). Arman et al. demonstrate that probes developed from iFGF/Nav PPI complexes can selectively modulate the activity of Na_v_s, highlighting potential avenues for targeted therapeutic development.

Na_v_1.8 is expressed in nociceptors within the peripheral nervous system (PNS) and plays a critical role in pain perception and transmission [6]. Plakhova et al. (Contribution 2) employ conformational analysis to examine how the binding of the polypeptide Ac-Lys-Lys-Lys-NH2 (Ac-KKK-NH2) to Na_v_1.8 reduces the effective charge transmitted by the channel and how this polypeptide can represent a promising analgesic candidate for potential application in human pain management. Ac-KKK-NH2 is shown to diminish pain responses in the formalin test, an established in vivo animal pain model.

In the third research article of this Special Issue, Festa et al. (Contribution 3) examine the interaction between a single-span type I transmembrane protein of previously unidentified function, Domain Family Member B (TMEM9B), and two neuronal endosomal chloride-transporting membrane proteins, ClC-3 and ClC-4. The nine-member CLC gene family is of considerable interest due to its role as chloride-transporting membrane proteins. The discovery of TMEM9B as a novel interaction partner for ClC-3 and ClC-4 provides insight that could significantly enhance our understanding of neuronal endosomal homeostasis. This finding establishes new directions for investigating both physiological and pathological mechanisms relevant to neuronal function and related diseases.

### 2.2. Review Articles

The reviews focus on various neurological disorders are listed below.

#### 2.2.1. Pain

Neuropathic and chronic pain are neurological disorders posing significant global healthcare challenges. Pain acts as a protective sensory mechanism but can become disabling when it develops into a disorder. Although many types of pain respond to current medications, chronic and neuropathic pain, as well as cancer-induced pain, often do not [7]. Additionally, common pain medications such as opioids may cause adverse effects, limited efficacy, and dependence and contribute to growing societal health, social, and economic problems [8]. Consequently, research is needed to find new therapeutic targets to develop pain medications. In their reviews, Felix et al. (Contribution 7) describe the role of various ion channel families in pain, while Yogi et al. (Contribution 9) detail the in vivo animal pain models and their use to test the involvement of ion channels in pain and ultimately the effectiveness of therapeutics targeted against ion channels.

Specifically, Felix et al. (Contribution 7) review neuropathic pain mechanisms, showing how dysregulation of Na_v_1.7 and Na_v_1.8 drives aberrant neuronal activity and sensitization. They highlight Ca_v_2.2 and the auxiliary protein CaVα2δ’s role in sustaining hyperexcitability and persistent pain, while K_v_7 channel dysfunction removes key inhibitory control. Overall, the review identifies ion channel dysregulation as central to neuropathic pain and points to K_v_ channel restoration as a promising therapeutic strategy.

On the other hand, Yogi et al. (Contribution 9) critically examine the challenge of selecting appropriate preclinical in vivo models to investigate ion channel–mediated pain, with particular focus on Na_v_1.7. Drawing on genetic evidence linking SCN9A mutations to familial pain disorders, they highlight the strong preclinical validation of Na_v_1.7 as a therapeutic target [9]. The authors underscore a persistent translational gap, as promising results from Nav1.7 blockade in animal pain models have not been replicated clinically. This review emphasizes the urgent need for more predictive, translationally relevant models and argues that targeting Na_v_1.7 alone is unlikely to yield effective analgesics. Instead, it advocates for the exploration of novel mechanisms alongside early and rigorous evaluation of pharmacokinetic, pharmacodynamic, and safety profiles to advance successful pain therapies.

#### 2.2.2. Alzheimer’s Disease

Rennie (Contribution 6) provides a focused review on α7 nicotinic acetylcholine receptors (α7nAChRs) in neurons, astrocytes, and microglia, highlighting how their expression and function shift in Alzheimer’s disease (AD). While α7nAChRs are implicated in cognitive processes and neuroinflammation, their roles vary by cell type and disease stage. The interaction between α7nAChR and amyloid-beta (Aβ) is complex, sometimes contributing to pathology and other times modulating receptor function. Despite promising preclinical studies using α7nAChR-targeting drugs, clinical trials have failed, in part due to the receptor’s diverse regulation and uncertain optimal therapeutic strategy. Rennie concludes that ongoing research into α7nAChR’s physiological and pathological roles is essential before effective AD treatments can be developed.

#### 2.2.3. Stroke and Glutamate Excitotoxicity

In the context of stroke, ion channels and ion channel receptors—NMDA receptors—play a critical role in mediating excitotoxicity, which contributes to neuronal injury and impedes recovery [10]. Glutamate-driven excitotoxicity is a central mechanism across acute and chronic neurological disorders, where excessive receptor activation disrupts ionic balance and triggers mitochondrial dysfunction, oxidative stress, and cell death. Amplified by impaired glutamate clearance and astrocytic dysfunction, these processes drive neuronal injury in stroke and major neurodegenerative diseases [11].

Cellott and Ballerini (Contribution 5) review extensively the glutamate-induced excitotoxicity, the role of glutamate receptors in excitotoxicity, the mechanism of excitotoxicity, and the role of excitotoxicity in stroke and chronic neurodegenerative diseases. The authors also present how nanotechnology can change the approach to neuroprotection, showing how graphene-based materials (GBMs), especially small graphene oxide nanosheets, alter pathological glutamate release and reduce inflammation while supporting neural compatibility. Their versatile roles, ranging from drug delivery to direct modulation of neurotransmission, position GBMs as innovative tools for tackling excitotoxic injury. However, standardization and rigorous testing are vital to ensure their safety and efficacy. Continued research integrating GBMs with cutting-edge methods such as CRISPR and machine learning may pave the way for personalized, responsive therapies that restore balance in neurodegenerative conditions.

Tauskela and Blondeau (Contribution 8) present a novel approach for selecting neuroprotective drugs in stroke, aiming to simplify and rationalize the process. Noting that all human trials to date have failed, the authors examine the methodological shortcomings of clinical trials assessing neuroprotective therapies for cerebral ischemia. Stroke affects one in four individuals globally and remains the second leading cause of death worldwide, with an annual mortality rate of approximately 5.5 million—a figure expected to climb as risk factors increase [12]. In their thought-provoking review, Tauskela and Blondeau highlight the ongoing failures in developing neuroprotective therapies for stroke, calling for a fundamental reconsideration of trial design and research priorities. They advocate for bold, efficacy-driven strategies—particularly those focusing on anti-excitotoxic interventions—while recognizing that more potent treatments may also carry greater risks. Their endorsement of intra-arterial drug delivery and personalized approaches based on stroke severity underscores a progressive vision: promote innovation in stroke treatment but carefully weigh safety concerns. This balanced perspective offers renewed optimism for advancing stroke care and breaking the cycle of disappointment.

#### 2.2.4. Leukodystrophies

Leukodystrophies (LDs) are a group of over fifty rare and progressive genetic diseases caused by damage in the myelin sheath (the insulating layer encasing nerve fibers). In a timely review, Belfiore et al. (Contribution 4) focus on ion channels as pivotal players in the pathophysiology of these disorders. Glial ion channels, found in oligodendrocyte precursor cells (OPCs), oligodendrocytes (OLs), astrocytes, and microglia, are important for the formation and maintenance of myelin [13]. The authors present how mutations in TMEM63A, ClC-2, and modulators like MLC1/GlialCAM cause specific forms of LDs, underscoring the importance of ionic and water homeostasis, as well as mechanosensitivity, in maintaining healthy myelin.

## 3. Conclusions

The nine contributions gathered in this Special Issue, “Ion channels and Neurological Disease—2nd Edition”, collectively reinforce the central role of ion channels and transporters in the regulation of neuronal excitability and the pathophysiology of a broad spectrum of neurological disorders. From molecular insights into channel structure and modulation to translational challenges in pain and stroke, these studies highlight both the progress achieved and the significant gaps that remain in the field. Importantly, the integration of multidisciplinary approaches emerges as essential for bridging the gap between mechanistic understanding and therapeutic application. As illustrated across the works included in this Special Issue, a deeper exploration of channel dynamics, their interactions with auxiliary proteins, and their context-dependent roles in disease will be critical to advancing precision medicine strategies. Ultimately, continued innovation in experimental design, model systems, and therapeutic targeting holds promise for overcoming current translational barriers. By refining our understanding of ion channel function in health and disease, future research may yield more effective, mechanism-based interventions capable of addressing the unmet clinical needs in neurological disorders.
